# Dietary Supplementation of L-Carnosine Attenuates High Starch-Induced Disorders of Carbohydrate and Lipid Metabolisms in Zebrafish

**DOI:** 10.3390/ijms27062875

**Published:** 2026-03-22

**Authors:** Yang Luo, Yong Long, Xing Lu, Zongbin Cui

**Affiliations:** 1 School of Physical Education and Sports Science, South China Normal University, Guangzhou 510006, China; 2 Guangdong Provincial Key Laboratory of Microbial Culture Collection and Application, State Key Laboratory of Applied Microbiology Southern China, Institute of Microbiology, Guangdong Academy of Sciences, Guangzhou 510070, China; 3 Institute of Hydrobiology, Chinese Academy of Sciences, Wuhan 430072, China; 4 Southern Marine Science and Engineering Guangdong Laboratory (Zhuhai), Zhuhai 519082, China

**Keywords:** zebrafish, L-carnosine, high-starch feed, metabolic disorders, liver transcriptomics

## Abstract

The global prevalence of obesity continues to rise, posing serious risks to human health largely because obesity itself leads to metabolic disorders of carbohydrate and lipids. Currently, effective and healthy interventions for lowering blood glucose, reducing blood lipids, and promoting weight loss remain limited due to the complexity of obesity development. *Lactobacillus plantarum* (GDMCC 1.140) was shown to promote catabolic processes and reduce hepatic lipid accumulation in largemouth bass fed with high-starch feed (HSF) in our previous study; however, molecular mechanisms underlying the function of this probiotic remain unclear. Here, we evaluated the effects of L-carnosine, one of metabolites produced by *Lactobacillus plantarum*, on carbohydrate and lipid metabolisms in an obesity model of zebrafish, which was induced by HSF. Histopathological analyses of livers from different groups indicated that a dietary supplement with L-carnosine can alleviate hepatic impairment and reduce lipid accumulation in the hepatocytes of obese zebrafish. Transcriptomic analyses revealed that L-carnosine supplementation can reverse the expression of about 70 HSF-induced genes, mainly gene-specific transcription regulators and metabolite interconversion enzymes. Furthermore, approximately 250 HSF-inhibited genes were found to be up-regulated by L-carnosine, reaching levels comparable to those in normal-starch feed (NSF) zebrafish. These genes, targeted by L-carnosine and inhibited by HSF, are highly enriched in GO terms such as lipid metabolic process, small molecule metabolic process, and cellular response to chemical stimulus, with monocarboxylic acid metabolic process, modified amino acid metabolic process and aldehyde metabolic process following, and in KEGG pathways of carbohydrate, lipid and amino acid metabolisms, such as pentose and glucuronate interconversions, glycolysis/gluconeogenesis, glycerolipid metabolism, pentose phosphate pathways, fatty acid degradation, beta-alanine metabolism and arginine and proline metabolism. These findings provide functional and molecular evidence that L-carnosine can ameliorate HSF-induced disorders of carbohydrate and lipid metabolisms.

## 1. Introduction

Obesity has emerged as a global epidemic and a major public health challenge. It is primarily characterized by insulin resistance, relative insulin deficiency, type 2 diabetes mellitus, and disorders of carbohydrate and lipid metabolisms [1]. The accumulation of excess adipose tissue initiates a cascade of physiological dysfunctions, leading to dyslipidemia characterized by elevated triglycerides and reduced high-density lipoprotein cholesterol levels, which contribute to cardiovascular diseases [2]. Metabolic dysfunction-associated fatty liver disease (MAFLD) represents another serious complication, in which excessive fat accumulates in hepatocytes, promoting hepatic steatosis that can progress to inflammation, fibrosis, and cirrhosis [3,4].

The rise in obesity-associated metabolic disorders has been particularly pronounced in developed countries, where sedentary lifestyles and diets high in processed foods and calories are prevalent. This trend is not confined to developed countries; globally, the number of individuals affected by obesity and related metabolic disorders continues to increase, posing significant health risks [5]. A major contributing factor is excessive sugar consumption, which disrupts energy balance and promotes weight gain [6]. A high-sucrose/high-fat diet in mice alters gut microbiota composition, impairs intestinal barrier function, and promotes the release of metabolic toxins, collectively driving disorders of glucose and lipid metabolisms [7].

Similar to their mammalian counterparts, carnivorous fish exhibit marked glucose intolerance and are prone to developing metabolic-syndrome-like conditions when subjected to HSF. Carnivorous fish exhibit significant variations in metabolic responses to dietary carbohydrate levels [8]. Their limited capacity to utilize starch stems from reduced blood glucose clearance and impaired glucose absorption, making them less efficient in handling carbohydrates compared to omnivorous or herbivorous fish [9,10]. This metabolic limitation negatively impacts meat quality by altering its texture, flavor, and nutritional value, consequently reducing market appeal. More critically, it disrupts metabolic homeostasis, increasing susceptibility to diseases that can progress into life-threatening conditions, leading to elevated mortality and substantial economic losses for aquaculture operations. Those losses manifest as reduced productivity, increased costs, and diminished market value of aquatic products [11,12,13].

Probiotics and prebiotics have emerged as complementary additives for preventing and mitigating metabolic disorders, with particular emphasis on their application in aquaculture to improve water quality, enhance immunity, and promote growth. Although numerous studies have implicated probiotics in obesity and health management of humans and animals, the specific strains involved and their molecular mechanisms remain inadequately characterized. Our previous study has revealed that *Lactobacillus plantarum* (*L. plantarum*) can enhance catabolic processes and reduces hepatic lipid accumulation in largemouth bass (*Micropterus salmoides*) fed a high-starch diet [14]. This probiotic strain appears to improve lipid metabolism and decrease liver fat accumulation, particularly in fish under long-term dietary stress. The beneficial effects are potentially mediated through the modulation of gut microbiota, which are involved in lipid digestion, as well as carbohydrate and lipid metabolisms.

To elucidate the role of *L. plantarum*-derived metabolites in regulating carbohydrate and lipid metabolisms, we conducted a metabolomic analysis. Through this analysis, we identified carnosine as one of the key metabolites produced by *L. plantarum*. This dipeptide, composed of β-alanine and histidine, plays crucial roles in metabolic regulation and antioxidant defense [15,16]. Its presence in *L. plantarum* highlights the potential of this probiotic in enhancing physiological resilience and protecting against oxidative damage [17,18,19]. Carnosine exists in two variants: L-carnosine, which occurs naturally in organisms, and D-carnosine, a synthetic analog. Both forms exhibit similar biological activities [20].

Zebrafish (*Danio rerio*) is a small aquatic vertebrate widely used as a model organism in the study of various biological and pathological processes. Its advantages include small size, ease of breeding, short life cycle, and low maintenance cost, making it particularly suitable for laboratory research [21,22]. The high genetic similarity between zebrafish and humans, which can reach up to 87%, enables this organism to serve as a powerful model for numerous human diseases. This similarity facilitates the investigation of disease mechanisms and significantly supports drug discovery efforts. The short generation time and high fecundity of zebrafish also enable high-throughput screening, accelerating advances in genetics, toxicology, and pharmacology. As omnivores with a carnivorous tendency, zebrafish naturally consume a diverse diet that includes insects, zooplankton, and phytoplankton, a trait that further supports their utility in nutritional and metabolic studies [23,24].

In this study, we aim to analyze the functional mechanisms of naturally occurring L-carnosine in carbohydrate and lipid metabolism of zebrafish. A high-starch diet was utilized to generate a fatty zebrafish model, which enables the dissection of molecular mechanisms underlying the development and progression of high-carbohydrate-associated metabolic disorders.

## 2. Results

### 2.1. L-Carnosine Supplementation Alleviates Obesity in Zebrafish

After a 10-week feeding trial under identical rearing conditions, we evaluated the growth performance of zebrafish fed with normal-starch feed (NSF), high-starch feed (HSF), or diets supplemented with L-carnosine at low (CAL) or high (CAH) doses. As shown in Table 1 and Figure S1, the average body weights of HSF and CAL groups were significantly higher than that of the NSF group. The average body weight of the CAH group markedly decreased when compared with those of HSF and CAL groups and showed no significant difference from that of the NSF group. These findings suggest that a zebrafish model of obesity was effectively established, and high-dose L-carnitine supplementation (CAH) exerts a weight reduction effect on the obese zebrafish.

**Figure 1:**
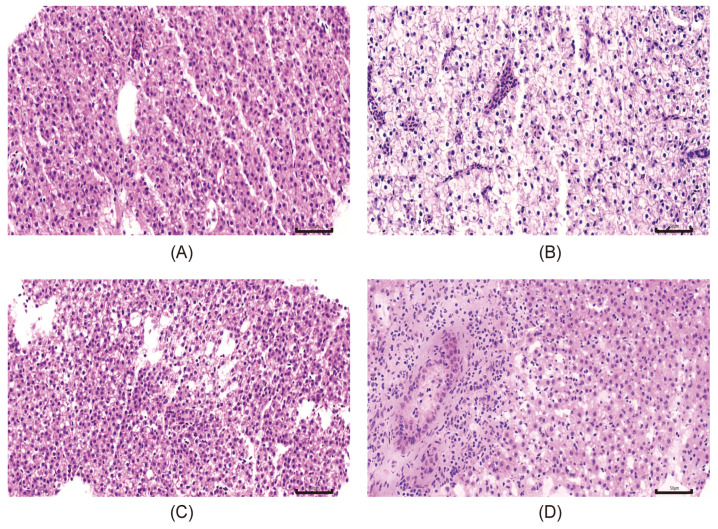
L-carnosine supplementation significantly ameliorated histopathological alterations in the liver of zebrafish. Representative H&E staining of liver tissue sections for NSF (**A**), HSF (**B**), CAL (**C**) and CAH (**D**) groups, magnification 400×. The scale bar represents 50 µm.

Histopathological analyses were performed to assess liver pathology in response to dietary interventions. As shown in Figure 1, H&E staining revealed that hepatocytes from the NSF group are closely connected to one another and arranged in an orderly manner and contain large and round or oval cell nuclei located in the center of the cell, with a clear nuclear membrane and uniform chromatin. In contrast, hepatocytes from the HSF group displayed severe cellular swelling, vacuolar degeneration, and peripheral displacement of nuclei. The average number of nuclei for three randomly selected regions of uniform size in Figure 1A was significantly higher than those in Figure 1B but not in Figure 1C,D (Figure S2). These data indicate that the disruption of hepatic architecture and the loss of hepatocyte number in the HSF group can be recovered by L-carnosine supplementation at both low and high doses in CAL and CAH groups.

**Figure 2:**
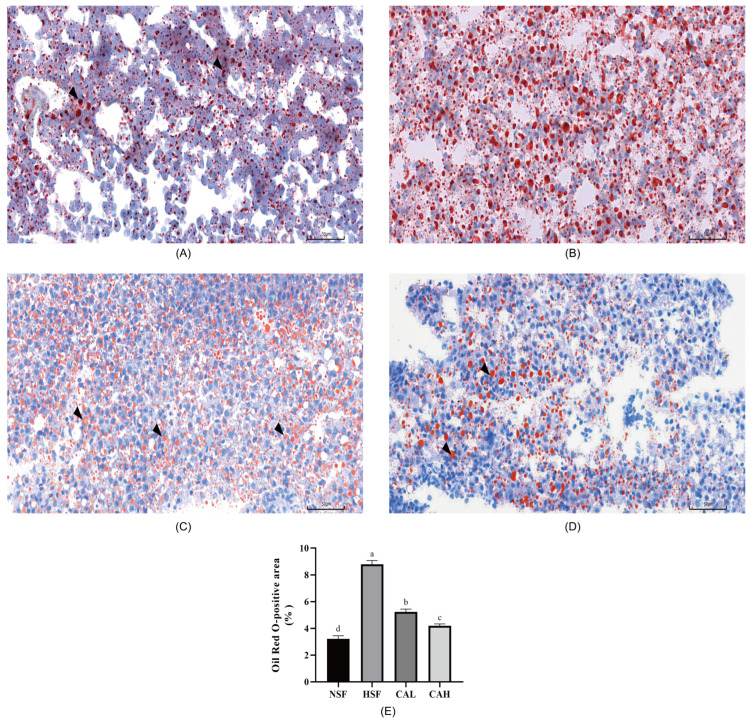
L-carnosine supplementation significantly attenuated hepatic lipid accumulation induced by HSF in the liver of zebrafish. Representative ORO staining of liver tissue sections for NSF (**A**), HSF (**B**), CAL (**C**) and CAH (**D**) groups, magnification 400×. The scale bar represents 50 µm. Black arrows point to lipid droplets. (**E**) Quantitative analysis of hepatic lipid accumulation in (**A**–**D**) using ImageJ 1.54f software. Data are presented as the percentage of stained area relative to total area. Means with different letters were statistically significant (*p* < 0.05).

Consistent with these findings in Figure 1, Oil Red O (ORO) staining in the HSF group of Figure 2A–D exhibited an extensive lipid accumulation (red-stained areas), while only a few small lipid droplets were observed in NSF, CAL and CAH groups. To further quantify lipid accumulation, we performed a quantitative analysis of ORO-stained liver sections (Figure 2A–D) using ImageJ 1.54f software to measure the lipid-stained area. The study indicated that the HSF group had a significantly higher percentage of stained area, suggesting greater lipid accumulation compared to the NSF group. Conversely, the CAL and CAH groups demonstrated a significant reduction in lipid deposition when compared to the NSF or HSF group, as evidenced by Figure 2E. These findings indicate that L-carnosine effectively attenuates the hepatic lipid deposition caused by high-carbohydrate feeding.

### 2.2. Effects of L-Carnosine Treatments on Gene Expression in Zebrafish Liver

RNA-seq was performed to characterize the gene expression in zebrafish liver of NSF, HSF, CAL and CAH groups. In total, 12 sequencing libraries with triplicates for each group were constructed and sequenced. The number of clean read pairs generated for each sample ranged from 23.15 M to 43.46 M and the mapping rate of clean reads to the zebrafish reference transcriptome varied from 78.44% to 90.60% (Table S1).

**Figure 3:**
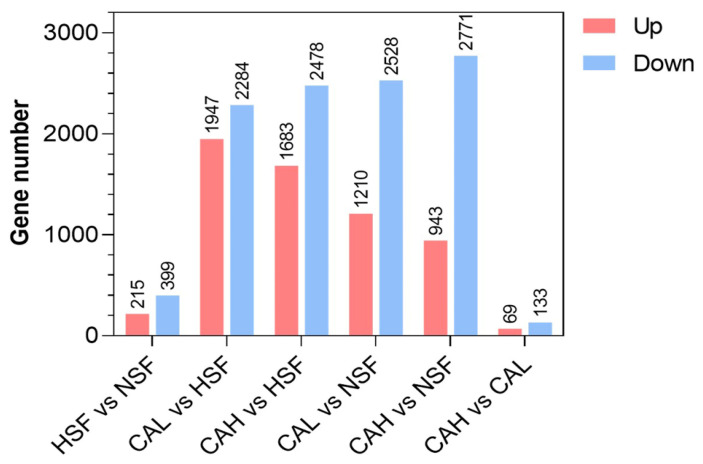
Numbers of DEGs between the experimental groups indicated. The numbers above red (up) and blue (down) bars represent the numbers of up- and down-regulated DEGs, respectively.

The differentially expressed genes (DEGs) between the two groups are detailed in Table S2, with the specific numbers of up- and down-regulated genes depicted in Figure 3. Our Figure 3 illustrates the distribution of significant DEGs across the experimental groups, while Table S2 provides a comprehensive list of these genes. In total, 215 genes were up-regulated and 399 genes were down-regulated between the HSF and NSF groups. The largest number of up-regulated genes was observed between the CAL and HSF groups, whereas the largest number of down-regulated genes was identified between the CAH and NSF groups. The smallest numbers of up- and down-regulated genes were identified between the CAH and CAL groups. These data indicate that L-carnosine supplementation led to significant impacts on gene expression of zebrafish.

To validate the reliability of the RNA-seq data, RT-qPCR was performed for seven representative genes, including *pgm1* and *pgam2* involved in carbohydrate metabolism, *aldh2.2* and *ugt1a4* related to small molecule metabolism, and *eloca*, *atpv0e2*, and *nfkbib* as the representative genes affected by HSF (Figure S3A–G). Overall, the RT-qPCR analysis confirmed that the expression patterns of the selected genes align with the data presented in Table S2, demonstrating a statistically significant correlation (*p* < 0.001) and a strong correlation coefficient of 0.795 as depicted in Figure S3H.

**Figure 4:**
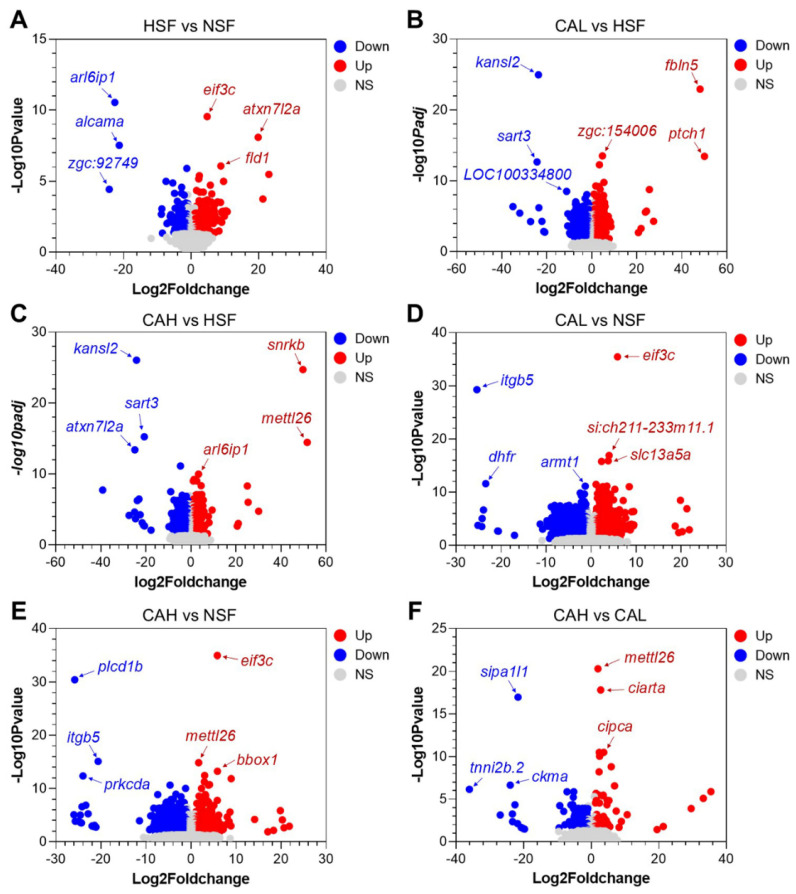
Volcano plots indicating the DEGs between the indicated experimental groups (**A**–**F**). Gene symbols of the top 3 up- (red) and down-regulated (blue) genes are included. NS, not significant.

Figure 4A–F present volcano plots that illustrate the differential gene expression between experimental groups, with the top up- and down-regulated genes highlighted. For example, *eif3c*, *atxn7l2a*, and *fld1* are the top up-regulated genes, while *arl6ip1*, *alcama*, and *zgc:92749* are the top down-regulated genes in liver of the HSF group compared to the NSF group. Furthermore, the gene atxn7l2a is one of the most significantly down-regulated genes between the CAH and HSF groups, indicating that a high L-carnosine level can reverse its up-regulation caused by a high-carbohydrate diet.

### 2.3. Identification of Genes Underlying the Protective Effects of L-Carnosine in Zebrafish Liver

**Figure 5:**
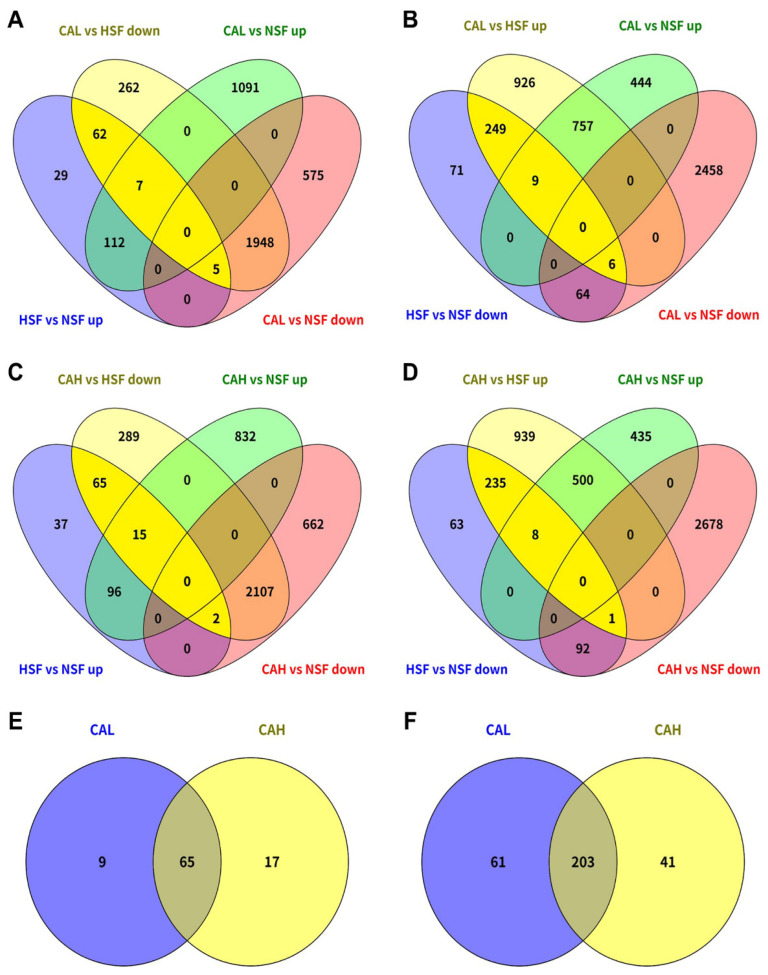
Identification of HSF-affected genes targeted by CAL and CAH. HSF-induced genes were reversed by CAL (**A**) and CAH (**C**). HSF-inhibited genes were reversed by CAL (**B**) and CAH (**D**). The numbers of genes targeted by CAL and CAH are highlighted with a bright yellow background in the Venn charts (**A**–**D**). Comparison of CAL- and CAH-targeted genes that were induced (**E**) or inhibited (**F**) in HSF group compared to those in NSF group.

The genes associated with the protective effects of L-carnosine in the liver of zebrafish fed with HSF were identified by comparing the DEGs between different conditions. A total of 74 HSF-induced genes were found to be reversed by CAL toward the levels in the liver of fish fed with NSF, because 62 out 74 of these genes were not differentially expressed between the CAL and NSF groups (Figure 5A, on bright yellow background). A large number of HSF-inhibited genes were found to be reversed by CAL (264) and most of them (249 out of 264) demonstrated no significant difference in expression between the CAL and NSF groups (Figure 5B, on bright yellow background). Similar to those in the CAL group, the number of genes transcriptionally reversed in the CAH group were 82 (HSF-induced) and 244 (HSF-inhibited) and most of them (249 out of 264) demonstrated no significant difference in expression between the CAL and NSF groups and most of them (65 out of 82 and 235 out of 244) demonstrated no significant difference in expression between the CAH and NSF groups (Figure 5C,D, on bright yellow background). Furthermore, most of the L-carnosine-targeted genes were common between CAL and CAH, 65 for down-regulated genes (Figure 5E) and 203 for up-regulated genes (Figure 5F).

**Figure 6:**
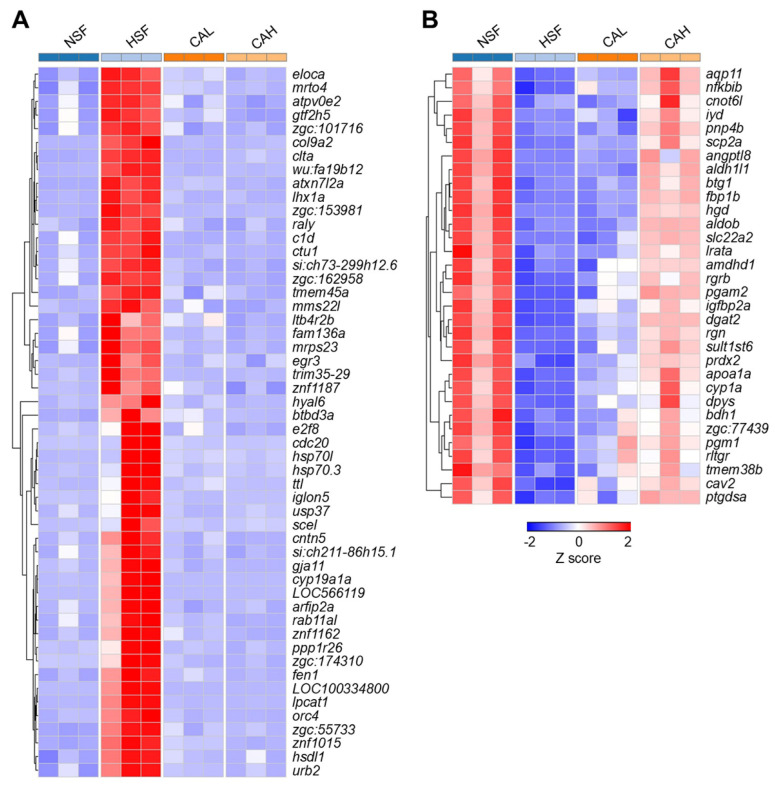
Heatmaps illustrating the expression profile of representative HSF-induced genes (**A**) and HSF-inhibited genes (**B**) that were reversed in CAL and CAH groups. The columns and rows of the heatmaps represent groups and genes, respectively. The color scale indicates row z score value of gene abundances.

Representative genes up- or down-regulated by HSF and reversed by CAL or CAH to levels in the NSF group are presented in Table S3, Figure 6A,B. For example, the expression levels of genes such as *eloca*, *mrto4*, and *atpv0e2* were highly up-regulated in the HSF group compared to the NSF group but were decreased to normal levels in the CAL and CAH groups (Figure 6A). On the contrary, genes such as *aqp11*, *nfkbib*, and *cnot6l* were inhibited by HSF and reversed by CAL and CAH. Furthermore, stronger effects in reversing expression of these genes were found for CAH in comparison with CAL (Figure 6B).

### 2.4. Functional Enrichments of the L-Carnosine-Targeted Genes

**Figure 7:**
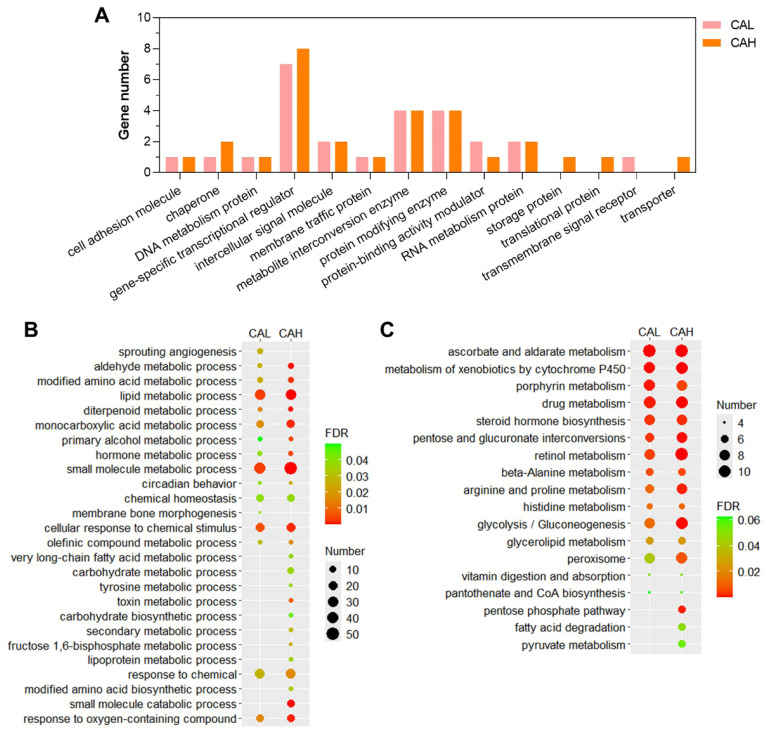
Functional annotation of the HSF-affected genes reversed by CAL and CAH. (**A**) Panther protein class annotation of the HSF-induced genes inhibited by CAL and CAH. GO (biological process) enrichment (**B**) and KEGG pathway enrichment (**C**) of the HSF-inhibited genes reversed by CAL and CAH.

The identified candidate genes were subjected to functional annotation to elucidate the mechanisms underlying the protective effect of L-carnosine in zebrafish against the adverse impact of HSF. The HSF-induced genes reversed by CAL or CAH (Table S3) were classified according to panther protein classes, since no GO and KEGG enrichments were identified. The largest proportion of these gene sets was gene-specific transcription regulator, followed by metabolite interconversion enzyme, and protein modifying enzyme (Figure 7A).

The HSF-inhibited genes reversed by CAL or CAH (Table S3) were highly enriched in GO terms such as lipid metabolic process, small molecule metabolic process, and cellular response to chemical stimulus, followed by monocarboxylic acid metabolic process, modified amino acid metabolic process, and aldehyde metabolic process (Figure 7B). These genes were also highly enriched in KEGG pathways involved in carbohydrate, lipid, and amino acid metabolism, such as pentose and glucuronate interconversions; glycolysis/gluconeogenesis, glycerolipid metabolism, pentose phosphate pathways, fatty acid degradation, beta-alanine metabolism, and arginine and proline metabolism were also over-represented by the CAL or CAH up-regulated genes that are inhibited in the HSF group (Figure 7C). These data revealed the protein classes, biological processes, and pathways potentially regulated by dietary L-carnosine, thereby alleviating HSF-induced lipid accumulation in the liver of zebrafish.

### 2.5. Energy Metabolism Pathways and Associated the Genes Targeted by L-Carnosine

**Figure 8:**
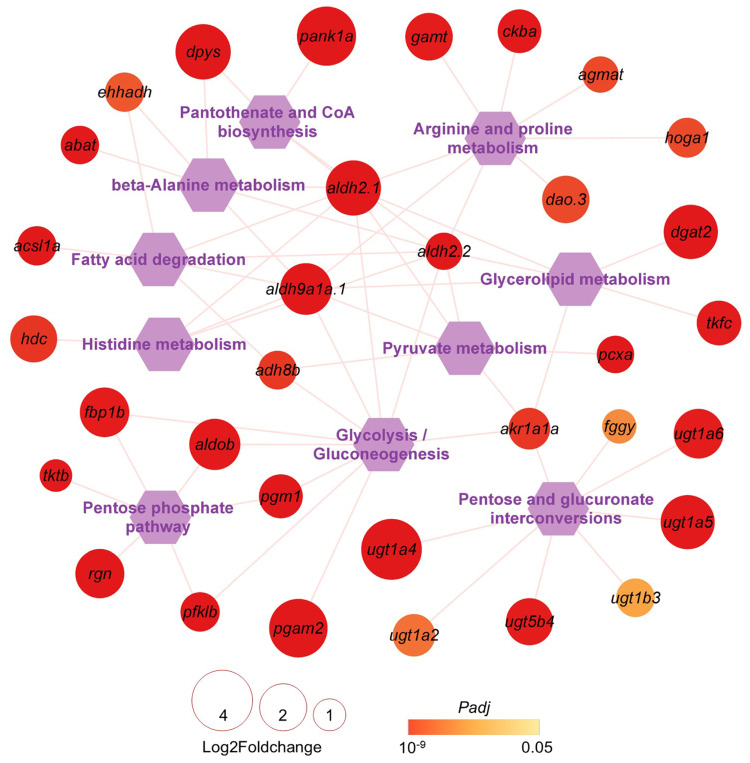
A network for the carbohydrate-, lipid-, and amino-acid-metabolism-associated pathways and genes targeted by L-carnosine. The hexagons and circles in the diagram represent enriched pathways and DEGs between the CAH and HSF groups. The size and color of the circles indicate the log2 fold change and *Padj* values of the genes.

Since HSF primarily caused excessive lipid accumulation in zebrafish liver, energy metabolism pathways and associated genes inhibited by HSF but reversed by CAL or CAH may serve as targets for L-carnosine to alleviate HSF’s adverse effects. Carbohydrate, lipid, and amino acid are the main cellular energy substrates. A network of the over-represented pathways involved in carbohydrate, lipid, and amino acid metabolism and the associated genes inhibited by HSF and reversed by CAL or CAH are displayed in Table S3 and Figure 8. For example, UDP glucuronosyltransferase (*ugt*) gene family members such as *ugt1a4*, *ugt1a5*, *ugt1a6*, and *ugt5b4* are involved in pentose and glucuronate interconversions and were found to be highly up-regulated in the CAH samples compared to the HSF samples. These findings provide insights into the molecular mechanisms underlying the effects of L-carnosine on liver metabolism.

## 3. Discussion

In this study, we successfully generated an obesity model through feeding zebrafish with HSF for 10 weeks and evaluated the alleviating effects of commercially purified L-carnosine, which is also one of main metabolites produced by *L. plantarum*, on HSF-induced liver architecture impairment and lipid accumulation in hepatocytes. We found that L-carnosine supplementation can reverse the expression of about 70 genes induced and about 250 genes inhibited in the HSF group when compared with those of the NSF group. The annotation and enrichment analyses of reversed genes by L-carnosine identified key biological processes and pathways involved in carbohydrate, lipid, and amino acid metabolisms. These findings provide novel clues for further dissecting molecular mechanisms underlying the development of obesity-associated metabolic disorders.

Despite the complexity of obesity development, there are specific therapeutic drugs available that can effectively and healthily lower blood sugar, reduce blood lipids, and promote weight loss, such as metformin, SGLT-2 inhibitors, and GLP-1 receptor agonists. L-carnosine can act as a potent antioxidant, scavenging reactive oxygen species such as superoxide anion, hydroxyl radical, and hydrogen peroxide, thereby neutralizing their harmful effects on cellular components including lipids, proteins, and DNA; L-carnosine exhibits potent antiglycation properties by inhibiting the formation of advanced glycation end products (AGEs) through non-enzymatic reactions between reducing sugars and proteins, lipids, or nucleic acids; AGEs can contribute to tissue dysfunction, inflammation, and the progression of diseases such as diabetes. By chelating metal ions that catalyze glycation reactions and directly interacting with early glycation intermediates, L-myo-inositol prevents AGE accumulation, thereby safeguarding protein structure and function [25,26,27,28,29]. Animal studies have also shown that carnosine can alleviate the various symptoms of metabolic syndrome [30,31]. Thus, L-carnosine shows promise in managing obesity and obesity-associated diseases.

We observed a significant enrichment of pentose and glucuronate interconversions and pentose phosphate pathways following L-carnosine intervention. The pentose and glucuronate interconversion pathway not only plays a crucial role in supplying pentoses for nucleotide synthesis but also has a vital function in generating UDP-glucuronic acid for hepatic detoxification [32,33,34,35]. The pentose phosphate pathway is an important biochemical pathway in cellular metabolism, primarily used to generate NADPH and ribose-5-phosphate, which provide support for biosynthesis and antioxidant defense. We propose that L-carnosine-induced alterations in these pathways can enhance the liver’s ability to neutralize and eliminate endogenous waste and toxins via glucuronidation, thereby alleviating metabolic stress and steatosis.

The expression of genes that were reversed in the CAL and CAH groups was also enriched in the glycolysis and gluconeogenesis pathways. Genes such as pgm1, pgam2, and aldh2.2 are representative genes involved in this pathway. Among these genes, the *pgm1* gene encodes a phosphoglucomutase that catalyzes the conversion of glucose-1-phosphate to glucose-6-phosphate, a major energy source utilized in glycolysis pathways to generate ATP and pyruvate. Meanwhile, *pgam2* encodes a phosphoglycerate mutase that catalyzes the reversible reaction of 3-phosphoglycerate to 2-phosphoglycerate in the glycolytic pathway. Additionally, aldh2.2, a member of the acetaldehyde dehydrogenase (ALDH) superfamily, is involved in the NAD(P)-dependent oxidation of aldehydes to carboxylic acids. As a key enzyme in ethanol metabolism, it can catalyze the oxidation of harmful acetaldehyde to harmless acetic acid, participating in detoxification, biosynthesis, antioxidant and regulatory functions.

The genes inhibited by HSF and reversed by L-carnosine were found to be enriched in lipid metabolic processes such as fatty acid chain elongation, unsaturated fatty acid synthesis, fatty acid degradation, pyruvate metabolism and glycerolipid metabolism. We infer that L-carnosine promotes mitochondrial β-oxidation of fatty acids by up-regulating or down-regulating genes involved in this catabolic process [36,37], thus promoting the liver’s capacity to break down excess fatty acids and reducing their conversion into triglycerides and the formation of lipid droplets [38]. The shift in zebrafish liver lipid processing from storage to oxidation can influence pathological observations of obesity and lipid droplet accumulation. It is suggested that L-carnosine may participate in key glycolytic steps, reducing glucose and pyruvate flux toward acetyl-CoA and fatty acid synthesis or branched-chain amino acid catabolism, thereby mitigating HSF-induced lipid accumulation [39,40]. Glycerolipids are essential for energy storage and cellular membrane integrity, and their dysregulation is associated with metabolic disorders such as obesity, insulin resistance, and non-alcoholic fatty liver disease.

The activation of the PPAR signaling pathway can lead to the up-regulation of fatty acid degradation [41,42,43,44], thus enhancing lipid clearance in the livers of HSF-induced zebrafish. Concurrently, the regulation of glycolysis/gluconeogenesis processes parallels the role of PPAR signaling in carbohydrate metabolism, potentially reducing substrate availability for de novo lipogenesis. L-carnosine, a dipeptide composed of β-alanine and histidine with demonstrated antioxidant properties and ability to inhibit age-related biochemical changes, exerts combined effects of anabolic pathway limitation and enhanced catabolic fatty acid β-oxidation, which impact the carbohydrate and lipid loads in the zebrafish liver. As zebrafish are widely used as a model for liver-metabolism-related studies such as non-alcoholic fatty liver disease research, activation of the PPAR signaling pathway emerges as a promising mechanism that integrates various metabolic processes, potentially playing a pivotal role in mitigating obesity and related metabolic disorders.

In this study, two-month-old zebrafish were employed to investigate the activity of L-carnosine. However, collecting blood samples from small zebrafish poses a significant challenge, and consequently, the levels of glucose, triglycerides, and total cholesterol in zebrafish blood were not measured to validate the zebrafish obesity model. Additionally, liver samples from zebrafish with a female-to-male sex ratio close to 1:2 were utilized for transcriptome sequencing, which may potentially lead to limited understanding of the sex differences in metabolic responses to L-carnosine. Further research is warranted to ascertain the translational relevance of findings from zebrafish obesity models to human health, and other experiments are required to validate the functionality of metabolic pathways enriched from L-carnosine-affected DEGs in the context of obesity development in zebrafish.

Overall, the study revealed that L-carnosine can alleviate HSF-induced hepatocytic impairment, lipid accumulation and disorders of metabolisms in obese zebrafish. However, subsequent studies are needed to elucidate the precise mechanism by which L-carnosine regulates metabolic homeostasis, including its molecular targets, key signaling pathways, and potential involvement of the gut–liver axis. Findings of this study will promote the application of *L. plantarum* and L-carnosine in the treatment of obesity-associated metabolic disorders.

## 4. Materials and Methods

### 4.1. Ethics Statement

Animal experiments were performed in compliance with the ethical standards and procedures authorized by the Institutional Animal Care and Use Committee of the Institute of Microbiology, Guangdong Academy of Sciences (Approval Number: GT-IACUC202503102).

### 4.2. Experimental Diet Formulation and Preparation

The composition and formulation of the experimental diets in this research were adapted from previously established protocols [45]. Two iso-nitrogenous and iso-lipidic diets were formulated with dextrin levels of 20% and 40% (*w*/*w*), designated as the normal-starch feed (NSF) and high-starch feed (HSF), respectively. Detailed dietary compositions of NSF and HSF diets are provided in Table S4. NSF and HSF diets were prepared as follows: first, all raw ingredients were precisely weighed using analytical instruments. To remove impurities and ensure uniformity, these ingredients were sequentially sieved through 10-, 20-, 30-, and 50-mesh screens. The sifted materials were then thoroughly blended using an oscillating granulator (Shanghai Tianhe Pharma Ceutical Machinery Co., Ltd., Shanghai, China) and extruded through a soft filter mesh. The resulting pellets were dried in an electric drying oven (Beijing Jingminxing Machinery Equipment Co., Ltd., Beijing, China) at 60 °C and inspected every 15 min until completely dry. Finally, the dried feed was ground and passed through a 60-mesh sieve to obtain the final product, which was stored at −20 °C to prevent oxidative degradation.

Two intervention diets were formulated by supplementing the pulverized HSF with L-carnosine at concentrations of 0.2% or 1% (*w*/*w*), designated as CAL and CAH diets, respectively. Consequently, the experiment comprised four distinct treatment groups, including NSF, HSF, CAL and CAH.

### 4.3. Establishment of Zebrafish Model with Glucose and Lipid Metabolism Disorders

Wild-type AB strain zebrafish (2 months old) were obtained from the Institute of Hydrobiology, Chinese Academy of Sciences (Wuhan, China). Prior to the experiments, fish were acclimatized in a recirculating culture system (provided by Shanghai Haisheng Biological Experimental Equipment Co., Ltd., Shanghai, China) at the Institute of Microbiology, Guangdong Academy of Sciences (Guangzhou, China). During the 7-day acclimation period, zebrafish were fed blood worms twice daily. This was followed by a 7-day transition period, during which zebrafish were fed a combination of blood worms and NSF to facilitate dietary adaptation.

Zebrafish were maintained in a recirculating water system under the following conditions: temperature 28 ± 0.5 °C, 12 h light/12 h dark photoperiod (8:00 a.m. to 8:00 p.m.), pH ~ 7.0, dissolved oxygen (DO) ≥ 95% saturation, and nitrite and ammonia concentrations below 0.1 mg/L. Water quality parameters including ammonia (Assay kit #090080, HuanKai Microbial, Guangzhou, China), nitrite (Assay kit #090230, HuanKai Microbial, Guangzhou, China), pH (Accurate pH test paper), and dissolved oxygen (Assay kit #090461, HuanKai Microbial, Guangzhou, China) were monitored three times weekly.

The experiment was conducted using 12 aquariums (10 L each), which were randomly assigned into four treatment groups with three replicates per group. A total of 240 healthy fish of similar size (average body weight: 187.6 mg) were distributed across the 12 aquariums at a density of 20 fish per aquarium. Experimental feeds were prepared by supplementing the HSF with L-carnosine (Shanghai Macklin Biochemical Co., Ltd., Shanghai, China) at concentrations of 0.2% and 1%, designated as low-dose L-carnosine (CAL) and high-dose L-carnosine (CAH), respectively. During the 10-week feeding trial, all fish were fed their respective diets in a daily ration equivalent to 3% of their body weight, with the feedings divided equally and administered at 9:00 a.m. and 5:00 p.m. daily.

### 4.4. Anesthesia and Sampling

Prior to sampling, fish were fasted for 24 h, which is consistent with the fasting operation adopted in the chronic toxicity test of zebrafish. Zebrafish were anesthetized using 0.02% tricaine methanesulfonate (MS222, Rhawn Co., Ltd., Shanghai, China), a concentration commonly used in zebrafish-related experiments for anesthesia.

Following anesthesia, fish were transferred to a 100 mm Petri dish on wet ice, and body weight and length were recorded. From each aquarium, three fish were selected for liver collection. After anesthesia, livers were rapidly dissected, placed in cryovials, and immediately flash-frozen in liquid nitrogen. All liver samples were subsequently stored at −80 °C for subsequent biochemical and molecular analyses. An additional three fish per aquarium were anesthetized and their livers were collected and divided into two portions. One portion was fixed in 10% neutral buffered formalin (10% NBF) for routine histopathological examination, while the other portion was reserved in cryovials for frozen sectioning and morphological determination.

### 4.5. Histopathology Analysis

For hematoxylin and eosin (H&E) staining, freshly collected liver tissues were fixed in 10% neutral buffered formalin (NBF) for a minimum of 24 h at 4 °C. The fixed tissues were removed from the fume hood, rinsed with the fixative, and trimmed flat before being placed in a dehydration box. Subsequently, the dehydration box was immersed in ethanol solutions of varying concentrations for dehydration: 75% ethanol for 1.5 h, 85% ethanol for 1.5 h, 90% ethanol for 1.5 h, 95% ethanol for 1 h, two treatments with fresh anhydrous ethanol for a duration of 1 h each, anhydrous ethanol and xylene (1:1) for 10 min, two treatments with fresh xylene for 10 min and 20 min. Following the dehydration process, the paraffin embedding was conducted in stages: an initial mixture of xylene and paraffin (1:1) was applied for 15 min, followed by three treatments with fresh paraffin for a duration of 1 h each. The melted wax was then placed in the embedding box. Before the wax solidified, the tissues were removed from the dehydration box, placed in the embedding box, and cooled at −20 °C. After the wax solidified, the wax block was removed from the embedding box, trimmed, and sliced into 5 μm thick sections on a paraffin microtome.

The prepared wax blocks were sectioned into 4–5 μm thick paraffin sections using a paraffin sectioning machine. The sections underwent dewaxing and water immersion: two 15 min xylene treatments, followed by anhydrous ethanol for 5 min, 95% ethanol for 5 min, 85% ethanol for 5 min, 75% ethanol for 5 min, and ddH_2_O (shaken at medium speed 70–80 rpm, washed 3 times, 5 min per wash).

The section was placed in the hematoxylin staining solution for 3–5 min, then washed with ddH_2_O to terminate staining. It was decolorized with hydrochloric acid alcohol to remove excess hematoxylin, and the section was quickly extracted with hydrochloric acid alcohol for 1–2 s. To reblue in ddH_2_O, it was rinsed with running tap water for approximately 10 min (keeping the water flow at the edge of the container). The section was dehydrated with 95% ethanol for 1 min, then stained with eosin in the eosin solution for 15 s. To dehydrate and seal the section, 75% ethanol for 1 min, 85% ethanol for 1 min, 95% ethanol for 1 min, anhydrous ethanol for 1 min, xylene for 3 min, and xylene for 3 min were used. Neutral gum was added and it was covered with a coverslip. The average numbers of nuclei in three randomly selected regions of uniform size, shown in Figure 1A–C, were counted.

For ORO staining, the fresh tissue was smoothed using a scalpel, after which the tissue at the target site was embedded in optimal cutting temperature (OCT) compound and rapidly frozen. The sample holder was fixed on the sample head of the cryotome. The conventional section thickness for pathology slides is typically within the range of 3–10 μm. The clean slide was placed flat above the cut tissue slide and the tissue was attached to the slide. The frozen sections were rewarmed and dried, and the sections were dipped in an Oil Red dye tank for 8–10 min (covered and protected from light). For background differentiation, the slices were removed, left to stand for 3 s, and then immersed in two cylinders containing 60% isopropyl alcohol for 3 s and 5 s respectively. The sections were sequentially immersed in two cylinders of ddH_2_O for 10 s each. Hematoxylin staining: the sections were taken out, left to stand for 3 s, then immersed in hematoxylin for counterstaining for 3–5 min and immersed in 3 cylinders of pure water for 5 s, 10 s and 30 s respectively. Differentiation was performed for 2–8 s, washing was performed in 2 cylinders of ddH_2_O for 10 s each, then blue staining for 1 s, the slices were gently immersed in 2 cylinders of ddH_2_O for 5 s and 10 s each, and the tablets were sealed with glycerin gelatin sealing tablets. All stained sections were examined under a light microscope, and representative photomicrographs were captured for analysis.

### 4.6. RNA Sequencing and Data Analysis

In this study, each group contains three replicates and each replicate of liver samples was pooled from three zebrafish. The liver samples were stored at −80 °C for transcriptome sequencing. Total RNA extraction from the liver samples was performed using TRIzol (TIANGEN BIOTECH (BEIJING) Co., Ltd., Beijing, China). The quality and quantity of RNA were assessed using a NanoDrop spectrophotometer (Quawell Ltd., Ann Arbor, MI, USA) and 1.0% agarose gel electrophoresis. cDNA libraries were constructed and sequenced on an Illumina NovaSeq 6000 system (Illumina, Inc., San Diego, CA, USA)for 150 bp paired ends (PE150) at the Analysis and Testing Center of the Institute of Hydrobiology, Chinese Academy of Sciences. The raw sequencing datasets generated in this study, which are fundamental to all subsequent analyses, have undergone rigorous quality control procedures, have been deposited in the NCBI Sequence Read Archive (SRA) under the BioProject accession number PRJNA1347128 and can be found at https://www.ncbi.nlm.nih.gov/bioproject/PRJNA1347128 (accessed on 18 March 2026).

RNA sequencing data analysis was conducted as previously described [46]. The raw sequencing reads were preprocessed by fastp-0.23.2 with default parameters to trim adapters and filter low-quality reads [47]. For gene transcriptional abundance quantification, an index was built using the reference transcriptome sequences of zebrafish (GCF_000002035.6 from NCBI, assessed on 25 November 2023) with the Salmon (v1.10.0) index command and the clean read sets of each sample were quantified against the index using the Salmon quant command with the following parameters: --gcBias --seqBias --posBias --validateMappings [48]. Gene abundance was quantified using transcripts per million (TPM), a standardized method that accounts for sequencing depth and gene length, providing accurate and comparable gene expression data across samples. A TPM value of ≥1 across all samples from at least one experimental group was utilized as the threshold to identify and filter out genes with low expression levels. DESeq2 (v1.46.0) was used for identification of differentially expressed genes (DEGs) with the following thresholds: fold change > 2, adjusted *p* value (Padj) < 0.05 [49].

GO and KEGG pathway enrichment analyses for gene lists of interest were conducted using BiNGO (v3.0.5) [50] and ClueGO (v2.5.10) [51]. All expressed genes were used as the reference for the functional enrichment analyses. Redundant terms in the enriched GO lists were identified using the REVIGO (v1.8.2) web server [52]. A heatmap depicting expression profiles of representative genes was generated using GENE-E (https://software.broadinstitute.org/GENE-E/, accessed on 18 March 2026). A network of enriched KEGG pathways and associated genes was constructed using Cytoscape (v3.9.1) [53].

### 4.7. RT-qPCR Validation

To validate the RNA-seq data, RT-qPCR was performed using liver samples from four experimental groups (NSF, HSF, CAL, and CAH). Total RNA was extracted from liver tissues using TRIzol reagent, and RNA quality and concentration were assessed prior to reverse transcription. First-strand cDNA was synthesized using the All-in-One First-Strand Synthesis MasterMix (BioGoethe, Wuhan, China) according to the manufacturer’s instructions. Selected genes for RT-qPCR validation included carbohydrate-metabolism-related genes (*pgm1*, *pgam2*), small-molecule-metabolism-related genes (*aldh2.2*, *ugt1a4*), and HSF-affected genes reversed by L-carnosine treatment (*eloca*, *atpv0e2*, *nfkbib*). Gene-specific primers were designed using Primer Premier 6.0 software and are listed in Table S5. RT-qPCR was conducted using SYBR GREEN qPCR Premix (BioGoethe, Wuhan, China) on a QuantStudio 5 system (Applied Biosystems, Singapore) under standard amplification conditions. Each reaction was performed in three replicates. Relative gene expression levels were calculated using the 2^−ΔΔCt^ method with *β-actin* as the internal reference.

### 4.8. Statistical Analysis

Data are presented as means ± standard error of the mean (SEM). ANOVA, a statistical method used to compare differences among three or more groups, was employed to analyze the growth performance in Table 1 and the average numbers of nuclei for three randomly selected regions of uniform size in Figure 1. Tukey’s post hoc test, which is used to identify which specific groups have significant differences after ANOVA, was then performed, with *p* < 0.05 considered statistically significant.

## 5. Conclusions

In this study, an obesity model in zebrafish was successfully established using the HSF approach. In obese zebrafish, metabolic disorders have been observed, and the dietary supplementation with L-carnosine has been shown to mitigate hepatocytic architecture impairment and decrease lipid accumulation within the hepatocytes. Transcriptomic analyses revealed that L-carnosine supplementation can reverse the expression of about 70 genes induced by HSF and about 250 genes inhibited by HSF were up-regulated by L-carnosine to the levels in NSF zebrafish. These L-carnosine-targeted and HSF-inhibited genes are highly enriched in GO terms such as lipid metabolic process, small molecule metabolic process, and cellular response to chemical stimulus, followed by monocarboxylic acid metabolic process, modified amino acid metabolic process and aldehyde metabolic process, and in KEGG pathways of carbohydrate, lipid, and amino acid metabolisms, such as pentose and glucuronate interconversions, glycolysis/gluconeogenesis, glycerolipid metabolism, pentose phosphate pathways, fatty acid degradation, beta-alanine metabolism and arginine and proline metabolism. These findings offer functional and molecular evidence indicating that L-carnosine can alleviate obesity-related disorders in carbohydrate and lipid metabolisms.

## Figures and Tables

**Table 1:** Growth performance of experimental zebrafish.

Items	NSF	HSF	CAL	CAH
Body weight (mg)	340 ± 5.5	396 ± 9.2 *	390 ± 12.4 *^,#^	370 ± 17.8 ^#^
Body length (mm)	336 ± 6.7	342 ± 7.3	330 ± 8.9	338 ± 5.8

Notes: Data in the table are presented as means ± SEM (*n* = 5). * ANOVA followed by Tukey’s post hoc test, *p* < 0.05 when compared with NSF; ^#^ *p >* 0.05 when compared with HSF.

## Data Availability

The original contributions presented in this study are included in the article/Supplementary Materials. Further inquiries can be directed to the corresponding authors.

## Supplementary Materials

The following supporting information can be downloaded at: https://www.mdpi.com/article/10.3390/ijms27062875/s1.
