# Supplementary material for: Dietary Supplementation of L-Carnosine Attenuates High Starch-Induced Disorders of Carbohydrate and Lipid Metabolisms in Zebrafish
Source: Int J Mol Sci. 2026 Mar 22;27(6):2875. doi: 10.3390/ijms27062875 (PMC13026341; doi:10.3390/ijms27062875)
Supplement: Supplementary file 1 [file ijms-27-02875-s001.zip › Figures S1, S2 and S3.docx]

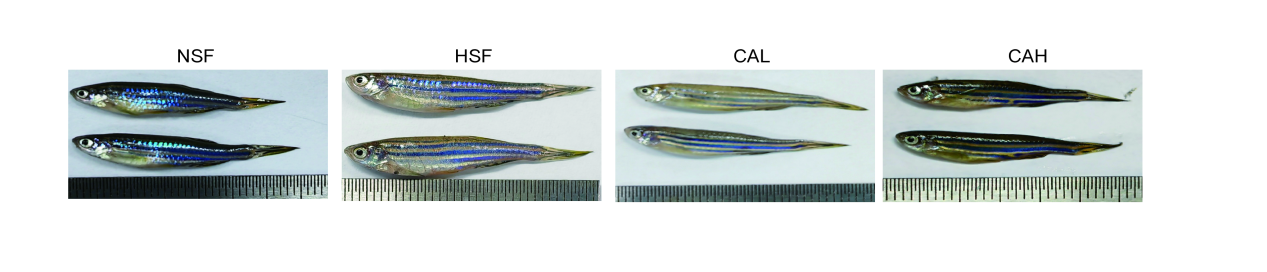


**Figure S1.** Representative images of two fish in NSF, HSF, CAL and CAH groups.


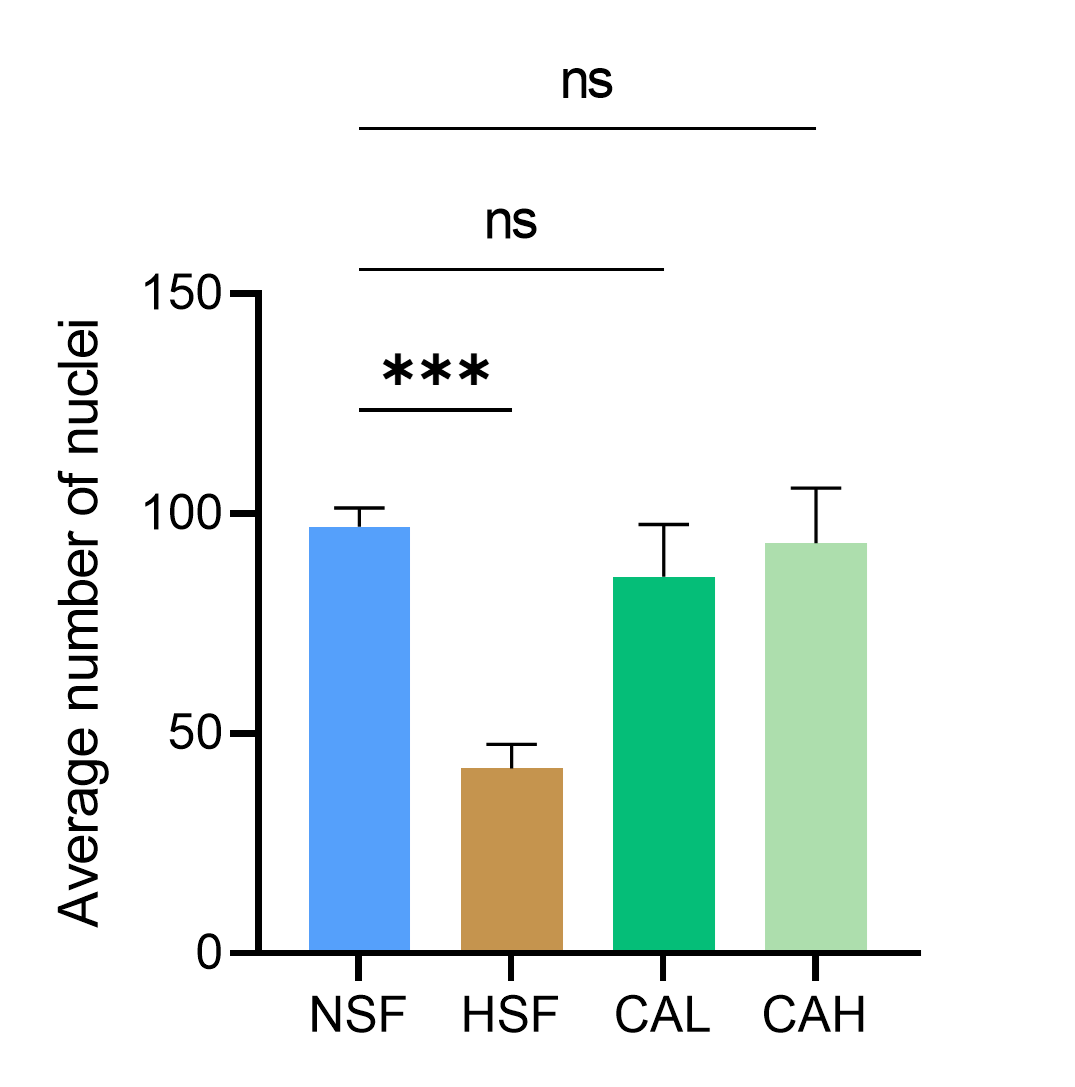


**Figure S2.** The average number of nuclei from three randomly selected regions of uniform size in Figure 1A-1D representing the NSF, HSF, CAL and CAH groups. Data are presented as means ± SEM (n=3). ANOVA analysis was performed and ***, p<0.01; ns, not significant.


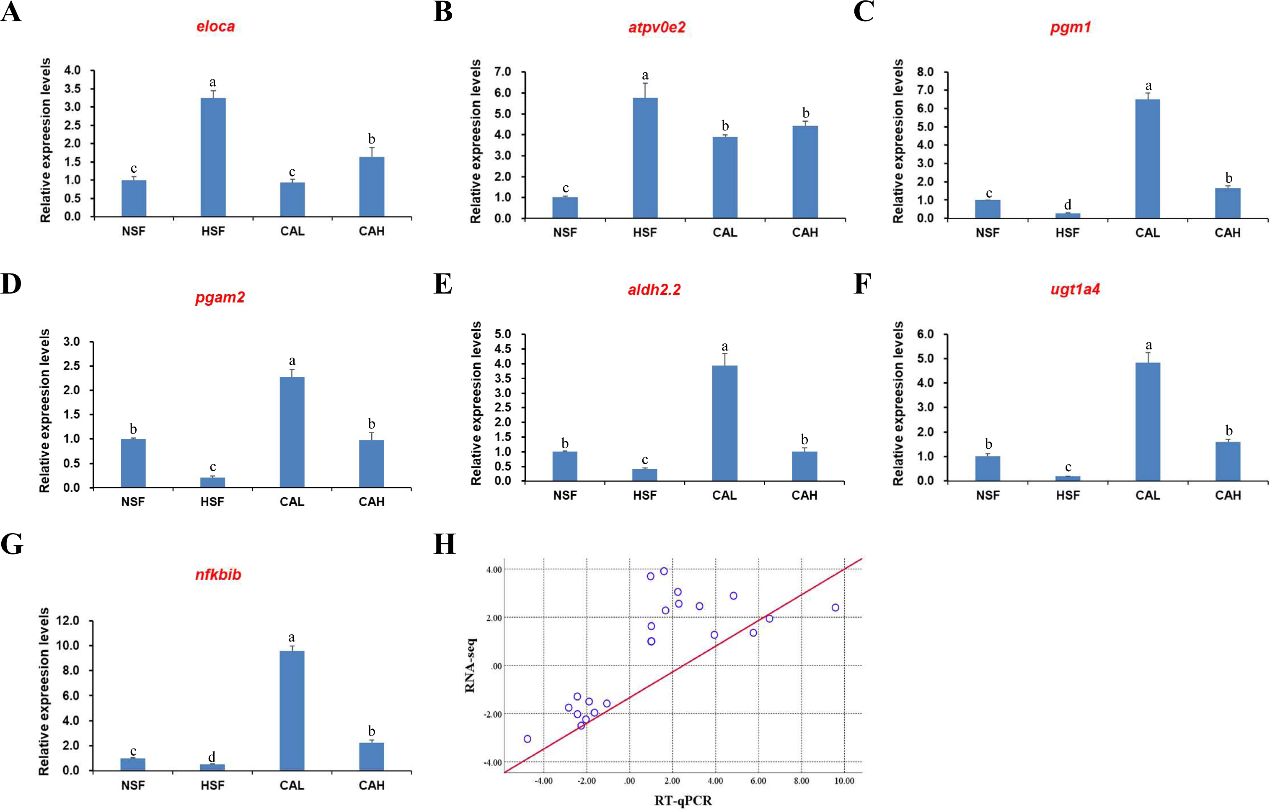


**Figure S3. RT-qPCR validation of representative differentially expressed genes identified by RNA-seq.** Relative expression levels of *eloca* (A), *atpv0e2* (B), *pgm1* (C), *pgam2* (D), *aldh2.2* (E), *ugt1a4* (F), and *nfkbib* (G) in zebrafish livers from NSF, HSF, CAL, and CAH groups. (H) Scatterplot for gene expressions between RNA-seq and RT-qPCR results, which is a recommended method for showing the correlation between the two datasets after normalization processing. The correlation was performed using Spearman rho test. Data are presented as means ± SEM. Means with different letters were statistically significant (*p* < 0.05).
