# Supplementary material for: Dietary Supplementation of L-Carnosine Attenuates High Starch-Induced Disorders of Carbohydrate and Lipid Metabolisms in Zebrafish
Source: Int J Mol Sci. 2026 Mar 22;27(6):2875. doi: 10.3390/ijms27062875 (PMC13026341; doi:10.3390/ijms27062875)
Supplement: Supplementary file 1 [file ijms-27-02875-s001.zip › Table S4-Formulation and proximate composition of the experimental diets.docx]

**Table S4.** Formulation and proximate composition of NSF and HSF diets (g/kg on a dry weight basis)

| Ingredients | NSF | HSF |
| --- | --- | --- |
| Casein | 380.0 | 380.0 |
| Gelatin | 8.0 | 8.0 |
| Soybean oil | 50.0 | 50.0 |
| Dextrin | 200.0 | 400.0 |
| Cellulose | 300.0 | 100.0 |
| Mineral premix^a^ | 10.0 | 10.0 |
| Choline chloride | 3.0 | 3.0 |
| Vitamin premix^b^ | 7.0 | 7.0 |
| Monocalcium phosphate | 30.0 | 30.0 |
| L-Threonine | 1.5 | 1.5 |
| L-Arginine | 8.5 | 8.5 |
| L-Tryptophan | 2.0 | 2.0 |
| L-carnosine | 0 | 0 |
| Crude protein^c^ | 314.4 | 315.2 |
| Crude lipid^d^ | 54 | 58 |
| Crude Fiber^e^ | 200 | 99 |
| Moisture^f^ | 39 | 46 |
| Ash^g^ | 39 | 37 |
| Total Sugars^h^ | 315 | 461.5 |
| Gross Energy (Kcal/100g)^i^ | 457 | 457 |
| Nitrogen-Free Extract^j^ | 354 | 445 |

^a^Mineral premix (mg/kg diet): CoCO_3_, 0.65; CuSO_4_·5H_2_O, 9.00; FeSO_4_·7H_2_O, 8.34; NaCl, 400.00; MgO, 240.00; MnSO_4_·H_2_O, 22.85; KI, 0.50; CaCO_3_, 1,860.00; ZnSO_4_·7H_2_O, 14.30; microcrystalline cellulose, 7,444.35.

^b^Vitamin premix (mg/kg diet): tocopherol acetate, 100; sodium menadione bisulfate, 25; retinyl acetate, 6.9; cholecalciferol,0.05; thiamin, 30; riboflavin, 30; pyridoxine, 20; cyanocobalamin, 0.1; nicotinic acid, 200; folic acid, 15; ascorbic acid, 1,000; inositol, 500; biotin, 3; calcium pantothenate, 100; microcrystalline cellulose 4,669.95.

Proximate composition detection method:

^c^Crude protein, GB/T 6432-2018 7.2;

^d^Crude lipid, GB/T 6433-2006(Type B),

^e^Crude Fiber, GB/T 6434-2022 5;

^f^Moisture, GB/T 6435-2014 8.1;

^g^Ash, GB/T 6438-2007;

^h^Total Sugars, DB12/T 847-2018;

^i^Gross Energy (Kcal/100g), Announcement No. 20 of the Ministry of Agriculture and Rural Affairs; ^j^Nitrogen-Free Extract, Announcement No. 20 of the Ministry of Agriculture and Rural Affairs.
