# Supplementary material for: Dietary Supplementation of L-Carnosine Attenuates High Starch-Induced Disorders of Carbohydrate and Lipid Metabolisms in Zebrafish
Source: Int J Mol Sci. 2026 Mar 22;27(6):2875. doi: 10.3390/ijms27062875 (PMC13026341; doi:10.3390/ijms27062875)
Supplement: Supplementary file 1 [file ijms-27-02875-s001.zip › Table S5. Primers used in RT-qPCR.docx]

**Table S5.** Primers used in RT-qPCR.

| **Primers** | **Forword (5’-3’)** | **Reverse (5’-3’)** | **NCBI No.** |
| --- | --- | --- | --- |
| *eloca* | ACCTCTGGCACCATCAAGGCTA | GCAGTTCCAGGGCAATCTCAGG | NM_001004673.1 |
| *atpv0e2* | AGTCGCAATGGTGTCGCACTC | TGACGCCTCTGTTCGGTCCTT | NM_001172635.1 |
| *pgm1* | TGTTGGCTCCTCTGGTGGACAT | AGGGCTAGTTTGGGCTGGATCA | NM_201025.1 |
| *pgam2* | CGCACCTGGCGTCTGAATGAA | GGCACGAGCAATGGTGTCCTT | NM_201024.1 |
| *aldh2.2* | CCGCTCACCGCACTGTATATCG | ATGGGACTGCTCCACTGCTTCT | NM_213301.3 |
| *ugt1a4* | TGCTCAGCGTTTGGTGTCTCG | ACAGCCAGGTCCAGTGGTTCA | NM_001177344.3 |
| *nfkbib* | ACAGACAGACAGGCGGTCACA | GAAGGAGGCACTGGACAACACC | NM_001128795.1 |
| *β-actin* | CGAGCAGGAGATGGGAACC | CAACGGAAACGCTCATTGC | NM_131031 |
